# Protective Role of *Loranthus regularis* against Liver Dysfunction, Inflammation, and Oxidative Stress in Streptozotocin Diabetic Rat Model

**DOI:** 10.1155/2020/5027986

**Published:** 2020-12-10

**Authors:** Ahmed Z. Alanazi, Faleh Alqahtani, Ramzi A. A. Mothana, Mohamed Mohany, Hatem M. Abuohashish, Mohammed M. Ahmed, Salim S. Al-Rejaie

**Affiliations:** ^1^Department of Pharmacology and Toxicology, College of Pharmacy, King Saud University, P.O. Box 55760, Riyadh-1145, Saudi Arabia; ^2^Department of Pharmacognosy, College of Pharmacy, King Saud University, P.O. Box 55760, Riyadh-1145, Saudi Arabia; ^3^Department of Biomedical Dental Sciences, College of Dentistry, Imam Abdulrahman Bin Faisal University, P.O. Box 1982, Dammam-31441, Saudi Arabia

## Abstract

Earlier studies revealed the potential therapeutic values of *Loranthus regularis* (*L. regularis*). This study evaluated *Loranthus regularis* (*L. regularis*) extract systemic antidiabetic effects and benefits against diabetic hepatocellular injuries through antioxidant and anti-inflammatory pathways using the streptozotocin (STZ) model in Wistar albino rats. After diabetes induction, animals were orally treated with *L. regularis* extract for 4 weeks. Serum levels of glucose, insulin, alanine aminotransferase (ALT), aspartate aminotransferase (AST), total cholesterol (TC), total triglycerides (TG), low-density lipoprotein (LDL), and high-density lipoprotein (HDL) were estimated. Furthermore, tumor necrosis factor alpha (TNF-*α*), interleukin-1 beta (IL-1*β*), interleukin-6 (IL-6), caspase-3, nitric oxide (NO), and prostaglandin E-2 (PGE-2) were estimated in serum. In liver, thiobarbituric acid reactive substances (TBARSs) and reduced glutathione (GSH) as well as the proinflammatory cytokines and enzymatic activities of superoxide dismutase (SOD), catalase (CAT), glutathione peroxidase (GPx), glutathione reeducates (GR), and glutathione-S-transferase (GST) were assayed. Finally, the degree of hepatic tissue damage was evaluated histologically. Treatment of the diabetic rats with *L. regularis* extract markedly reduced the elevated serum levels of glucose, ALT, AST, TC, TG, LDL, TNF-*α*, IL-1*β*, IL-6, caspase-3, NO, and PGE-2. *L. regularis* extract also improved serum levels of insulin and HDL. The elevated TBARS, TNF-*α*, IL-1*β*, and IL-6 levels in hepatic tissue of diabetic animals were reduced by *L. regularis*. Moreover, *L. regularis* extract significantly restored the diminished hepatic GSH level and enzymatic activities of SOD, CAT, GPx, GR, and GST in diabetic animals. The biochemical protective effects of *L. regularis* were associated with improved histological hepatocellular integrity and architecture. Taken together, *L. regularis* has therapeutic effects against diabetic-induced hepatic complications. The restored liver functions and cellular damage might be mediated through free radicals scavenging and proinflammatory cytokine inhibition.

## 1. Introduction

Diabetes mellitus (DM) is a chronic metabolic disorder, with a global prevalence among all ages. DM occurrence is escalating every year, which makes it a public health problem [1]. It is also reported that DM is associated with several serious complications that affects most of the physiological systems including the liver, neurons, heart, and kidneys due to persistent hyperglycemia and glucose intolerance [2].

Diabetic-associated hepatotoxicity is major complication of DM. Studies have found that DM could be considered as a risk factor for nonalcoholic fatty liver disease [3]. In addition, hyperglycemia was reported to exaggerate acute liver injury through inflammasome activation [4]. The deficiency of intracellular glucose in hepatic cells during DM results in damaging and necrotic effects, which stimulate apoptotic response [5]. Furthermore, several hepatic inflammatory responses were reported in reaction to DM [6]. Oxidative stress and the associated formations of reactive oxygen species (ROS) are considered the major causative factor in the pathogenesis of diabetic-induced hepatotoxic injuries [7]. During this event, the intracellular generation of free radicals exceeds the antioxidant capacity of hepatocytes leading to lipid peroxidation and oxidative necrosis. Moreover, the hepatocyte injury results in complex biological events that include neutrophil infiltration and activation of Kupffer cells. Such response stimulates apoptotic pathways and the release of inflammatory cytokines [8].

Nowadays, the use of medicinal plants has gained more intention as they possess several pharmacological actions owing to their biologically active constituents. *L. regularis* is a traditional medicinal plant used widely throughout Asian countries such as Yemen and Saudi Arabia and some African countries such as Ethiopia. *L. regularis* belongs to family Loranthaceae. It was shown that *L. regularis* could grow on several trees such as *Zizyphus spina-christi*, *Ficus*, and *Acacia* sp. *L. regularis* is considered a hemiparasitic shrub as it takes its water and mineral requirements from these trees, while carbohydrates are photosynthesized in its green leaves [9]. *L. regularis* is also known as a member of mistletoe. Several pharmacological studies have identified the therapeutic value of many mistletoe species in traditional medicine including antihypertensive, antidiabetic, and anti-inflammatory [10]. Furthermore, it was revealed that the active constituents of *L. regularis* could act as antioxidant and antimicrobial agents with a moderate anticancer activity [11]. Mothana et al. found that the phenolic constituents of *L. regularis* are effective against inflammation, nociception, fever, and oxidative stress [10]. These findings suggest the potential beneficial role of *L. regularis* against metabolic diseases, where oxidative stress and inflammation are deemed as causative factors.

To date, no other articles devoted to pharmacological properties of *L. regularis* have been published. On the basis of the previously found strong antioxidant activity of *L. regularis* and in continuation of our ongoing research [10] for bioactive compounds from interesting medicinal Yemeni plants, *L. regularis* was selected in this study. The main objectives of this study were to evaluate the potential protective effects of *L. regularis* against hyperglycemia and diabetic-induced hepatocellular damage and to assess its antioxidative stress and anti-inflammatory effects as mechanisms of action.

## 2. Materials and Methods

### 2.1. Animals

Twenty-four male Wistar albino rats weighing in between 250 and 270 g were received from Experimental Animal Care Center at College of Pharmacy, King Saud University (KSU). They were maintained under controlled conditions of temperature (22 ± 1°C), humidity (50–55%), and light (12 h light/12 h dark cycle). Before the start of the experiment, animals were acclimatized to the laboratory conditions for 7 days. They had free access to purina rat chow and drinking water. All experimental procedure including euthanasia was conducted in accordance with the National Institute of Health Guide for the Care and Use of Laboratory Animals, Institute for Laboratory Animal Research (NIH Publication No. 80-23; 1996). Ethical approval (SE-19-146) was obtained from vice-rector office of graduate studies and scientific research, KSU, Riyadh, Kingdom Saudi Arabia.

### 2.2. Diabetes Induction

Experimental diabetes was induced by a single intraperitoneal injection of 60 mg/kg freshly prepared streptozotocin (STZ; Sigma-Aldrich, ST. Louis, MO, USA) in 0.1 mol/L citrate buffer (pH 4.5), while control animals received equal volume of plain citrate buffer [12]. 72 hr following STZ injection, DM was verified by estimation of fasting blood glucose levels from animal's tail vein using Accu-Check Compact-Plus glucose meter system (Roche Diagnostics, Meylan, France) [13]. Animals with a glucose level >13.9 mMol/L were considered diabetic.

### 2.3. Plant Material and Extraction

Collection and extraction of the plant materials were conducted as described before [14]. Aerial parts including leaves, flowers, and twigs of *L. regularis* was collected from Al-Mahwit governate (Yemen) and identified at the pharmacognosy department, Faculty of Pharmacy, Sana'a University. Voucher specimen (Mo-M05) was deposited at the pharmacognosy Department, Faculty of Pharmacy, Sana'a University. One kilogram of air-dried and grinded plant material was extracted with 3000 ml methanol (CH_3_OH) for 5 hours by utilizing a Soxhlet apparatus. The obtained methanol extract was filtered and evaporated by using a rotary evaporator to give the crude methanol extract (114 g). In our previous work, we conducted complete extraction, fractionation, and isolation methodology for *L. regularis*. These procedures resulted in separation and purification of three biologically active flavonoids: quercetin 3-O-b-D-galactopyranoside, quercetin 3-Ob-L-arabinopyranoside, and quercetin 3-Oa-L-rhamnopyranoside [10].

### 2.4. Study Design

Animals were divided in to four groups by taking six rats in each as follows: (1) control animals treated with vehicle (control), (2) diabetic rats treated with vehicle (STZ), (3) diabetic rats treated with *L. regularis* 150 mg/kg/day (Lr (150)), and (4) diabetic rats treated with *L. regularis* 300 mg/kg/day (Lr (300)). *L. regularis* extract was suspended in 0.25% carboxymethyl cellulose sodium (CMC) solution and given orally once a day by gavage for 4 weeks starting one week after STZ injection [10]. An equal volume of CMC solution was given to control and STZ groups during the treatment period. Blood samples were collected through cardiac puncture under anesthesia induced by intraperitoneal injection of ketamine (92 mg/kg, Hikma Pharmaceuticals, Amman, Jordan) and xylazine (10 mg/kg, Bayer, Turkey) mixture. The blood samples were centrifuged at 1800 RCF for 10 min. Serum samples were labeled and stored at −20°C until analysis. Finally, animals were decapitated and immediately liver samples were dissected. Cross sections of liver samples were preserved 10% formalin for histopathology, and the remaining tissues were dipped in liquid nitrogen for a minute then stored at −80°C until analysis.

### 2.5. Serum Analysis

In serum, the levels of glucose, insulin, alanine aminotransferase (ALT), aspartate aminotransferase (AST), total cholesterol (TC), total triglycerides (TG), low-density lipoprotein (LDL), and high-density lipoprotein (HDL) were estimated using commercially available diagnostic kits (Human, Wiesbaden, Germany). Moreover, the concentrations of proinflammatory biomarkers were estimated using diagnostic kits (R&D systems Inc., USA). Tumor necrosis factor alpha (TNF-*α*), interleukin-1 beta (IL-1*β*), interleukin-6 (IL-6), and prostaglandin E-2 (PGE-2) were estimated using rat ELISA kits (Catalog #RTA00, RLB00, R6000B, and KGE004B; respectively). Caspase-3 was assayed using colorimetric assay kit (Catalog #K106-100), while nitric oxide (NO) was determined using total nitric oxide and nitrate/nitrite assay.

### 2.6. Tissue Analysis

Liver portions were homogenized in physiological buffer (1 : 10, w/v). Thiobarbituric acid reactive substances (TBARS) and reduced glutathione (GSH) levels were measured using diagnostic kits (Cayman Chemical Co., USA). In postmitochondria supernatants of hepatic tissues, the enzymatic activities of superoxide dismutase (SOD), catalase (CAT), glutathione peroxidase (GPx), glutathione reeducates (GR), and glutathione-S-transferase (GST) were determined by assay kits (R&D systems Inc., USA).

### 2.7. Histopathological Procedures

A cross-sectional portion of liver tissues from each group was preserved in 10% buffered formalin. The samples were embedded in paraffin blocks, and sections of 5 *μ*m thickness were cut using a Leica CM3050 S Research Cryostat (Leica Biosystems, USA). The sections were stained with hematoxylin and eosin (H&E). Finally, they were examined under the microscope for histopathological changes by an observer who was blind with respect to the treatment groups. The extent of hepatic necrosis and inflammation were semiquantitatively scored as follows: (0) no inflammatory or necrotic foci, (1) less than 2 inflammatory or necrotic foci, (2) between 2 and 4 inflammatory or necrotic foci, and (3) more than 4 inflammatory or necrotic foci. The mean score of each group was calculated and determined on five randomly chosen fields.

### 2.8. Statistical Analysis

The mean ± standard error of the mean (SEM) of each group was statistically compared to all other groups using one-way analysis of variance (ANOVA) followed by the Student–Newman–Keuls multiple comparison test (*n* = 6). The statistical variability between groups was considered significant when *p* values were ≤0.05. GraphPad Prism software, version 5 (GraphPad Software, Inc., La Jolla, CA, USA), was employed as statistical software.

## 3. Results

**Figure 1:**
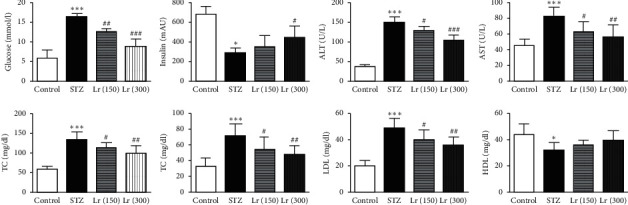
Effects of *L. regularis* (Lr) on diabetic-induced changes in serum glucose, insulin, alanine aminotransferase (ALT), aspartate aminotransferase (AST), total cholesterol (TC), total triglycerides (TG), low-density lipoprotein (LDL), and high-density lipoprotein (HDL) levels. Data were expressed as mean ± SEM (*n* = 6) and analyzed using one-way ANOVA followed by Student–Newman–Keuls as a post hoc test. ^*∗*^Control *vs* STZ group;  ^#^STZ *vs* Lr (150) or Lr (300). *P* values are considered significant when  ^*∗*^ or  ^#^*P* < 0.05, ^*∗∗*^ or  ^##^*P* < 0.011, and ^*∗∗∗*^ or  ^###^*P* < 0.001.

Experimentally induced diabetes in rats significantly (*p* ≤ 0.001) elevated blood glucose, ALT, and AST levels, while diminished insulin level (*p* ≤ 0.05), in STZ group as compared with the nondiabetic control group. Treatment of the diabetic rats with *L. regularis* in 150 and 300 mg/kg doses significantly reduced the blood glucose (*p* ≤ 0.01 and *p* ≤ 0.001, respectively), ALT (*p* ≤ 0.05 and *p* ≤ 0.001, respectively), and AST (*p* ≤ 0.05 and *p* ≤ 0.01, respectively) levels, while only the 300 mg/kg dose significantly (*p* ≤ 0.05) elevated insulin levels, as compared with the STZ group. TC, TG, and LDL were significantly (*p* ≤ 0.001) increased, while HDL was significantly (*p* ≤ 0.05) reduced, in serum of diabetic-untreated rats as compared with nondiabetic group. Both doses of *L. regularis* significantly (*p* ≤ 0.05 and *p* ≤ 0.01, respectively) decreased the serum levels of TC, TG, and LDL in diabetic animals as compared with the STZ group (Figure 1).

**Figure 2:**
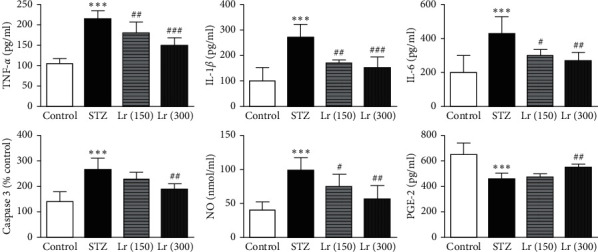
Effects of *L. regularis* (Lr) on diabetic-induced changes in serum tumor necrosis factor-*α* (TNF-*α*), interleukin-1*β* (IL-1*β*), interleukin-6 (IL-6), caspase-3, nitric oxide (NO), and prostaglandin E-2 (PGE-2) levels. Data were expressed as mean ± SEM (*n* = 6) and analyzed using one-way ANOVA followed by Student–Newman–Keuls as a post hoc test. ^*∗*^Control *vs* STZ group;  ^#^STZ *vs* Lr (150) or Lr (300). *P* values are considered significant when  ^*∗*^ or  ^#^*P* < 0.05, ^*∗∗*^ or  ^##^*P* < 0.01, and ^*∗∗∗*^ or  ^###^*P* < 0.001.

There was a significant (*p* ≤ 0.001) augmented serum expressions of inflammatory markers including TNF-*α*, IL-1*β*, IL-6, caspase-3, NO, and PGE-2 in diabetic-untreated animals as compared with the nondiabetic group. *L. regularis* treatment in 150 mg/kg dose significantly decreased the serum expressions of TNF-*α* (*p* ≤ 0.01), IL-1*β* (*p* ≤ 0.01), IL-6 (*p* ≤ 0.05), and NO (*p* ≤ 0.05) as compared with the STZ group. The higher *L. regularis* dose (300 mg/kg) significantly reduced the serum expressions of TNF-*α* (*p* ≤ 0.001), IL-1*β* (*p* ≤ 0.001), IL-6 (*p* ≤ 0.01), caspase-3 (*p* ≤ 0.01), NO (*p* ≤ 0.01), and PGE-2 (*p* ≤ 0.05) in diabetic rats as compared with the STZ group (Figure 2).

**Figure 3:**
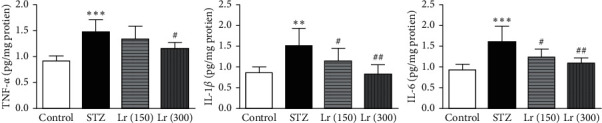
Effects of *L. regularis* (Lr) on diabetic-induced changes in hepatic proinflammatory cytokines including tumor necrosis factor-*α* (TNF-*α*), interleukin-1*β* (IL-1*β*), and interleukin-6 (IL-6) levels. Data were expressed as mean ± SEM (*n* = 6) and analyzed using one-way ANOVA followed by Student–Newman–Keuls as a post hoc test. ^*∗*^Control *vs* STZ group;  ^#^STZ *vs* Lr (150) or Lr (300). *P* values are considered significant when  ^*∗*^ or  ^#^*P* < 0.05, ^*∗∗*^ or  ^##^*P* < 0.01, and ^*∗∗∗*^ or  ^###^*P* < 0.001.

In liver tissues of diabetic animals, the expressions of inflammatory cytokines such as TNF-*α* (*p* ≤ 0.001), IL-1*β* (*p* ≤ 0.01), and IL-6 (*p* ≤ 0.001) were significantly elevated as compared with the nondiabetic group. *L. regularis* 150 mg/kg dose significantly (*p* ≤ 0.05) restored the hepatic expressions of IL-1*β* and IL-6. The 300 mg/kg dose of *L. regularis* significantly repaired the hepatic expression of TNF-*α* (*p* ≤ 0.05), IL-1*β* (*p* ≤ 0.01), and IL-6 (*p* ≤ 0.01) in diabetic animals as compared with the diabetic-untreated STZ group (Figure 3).

**Figure 4:**
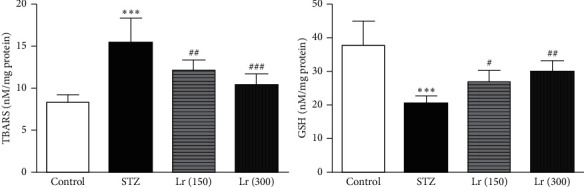
Effects of *L. regularis* (Lr) on diabetic-induced changes in hepatic thiobarbituric acid reaction substances (TBARs) and glutathione (GSH) levels. Data were expressed as mean ± SEM (*n* = 6) and analyzed using one-way ANOVA followed by Student–Newman–Keuls as a post hoc test. ^*∗*^Control *vs* STZ group;  ^#^STZ *vs* Lr (150) or Lr (300). *P* values are considered significant when  ^*∗*^ or  ^#^*P* < 0.05, ^*∗∗*^ or  ^##^*P* < 0.01, and ^*∗∗∗*^ or  ^###^*P* < 0.001.

The lipid peroxidation marker, TBARS, was significantly (*p* ≤ 0.001) augmented, while the endogenous antioxidant, GSH, was significantly (*p* ≤ 0.001) repressed in diabetic animals as compared with the control group. Both doses of *L. regularis* (150 and 300 mg/kg) significantly inhibited TBARS level (*p* ≤ 0.01 and *p* ≤ 0.001, respectively) and raised GSH concentration (*p* ≤ 0.05 and *p* ≤ 0.01, respectively) in hepatic tissues from diabetic animals as compared with the STZ group (Figure 4).

**Figure 5:**
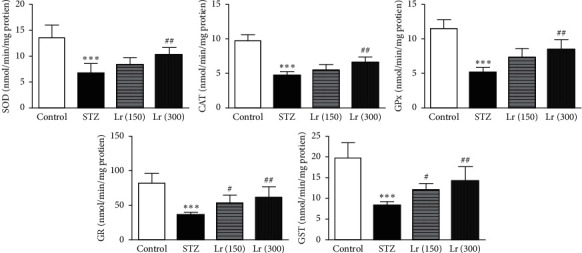
Effects of *L. regularis* (Lr) on diabetic-induced changes in hepatic enzymatic activities including superoxide dismutase (SOD), catalase (CAT), glutathione peroxidase (GPx), glutathione reductase (GR), and glutathione-S-transferase (GST). Data were expressed as mean ± SEM (*n* = 6) and analyzed using one-way ANOVA followed by Student–Newman–Keuls as a post hoc test. ^*∗*^Control *vs* STZ group;  ^#^STZ *vs* Lr (150) or Lr (300). *P* values are considered significant when  ^*∗*^ or  ^#^*P* < 0.05, ^*∗∗*^ or  ^##^*P* < 0.01, and ^*∗∗∗*^ or  ^###^*P* < 0.001.

The hepatic enzymatic activities of SOD, CAT, GPx, GR, and GST were significantly (*p* ≤ 0.001) repressed in diabetic-untreated animals as compared with the control group. *L. regularis* treatment in 150 mg/kg dose was able to increase the hepatic enzymatic activities of GR and GST significantly (*p* ≤ 0.05). However, the 300 mg/kg dose of *L. regularis* significantly (*p* ≤ 0.01) increased the hepatic enzymatic activities of SOD, CAT, GPx, GR, and GST in diabetic animals as compared with the diabetic-untreated STZ group (Figure 5).

**Figure 6:**
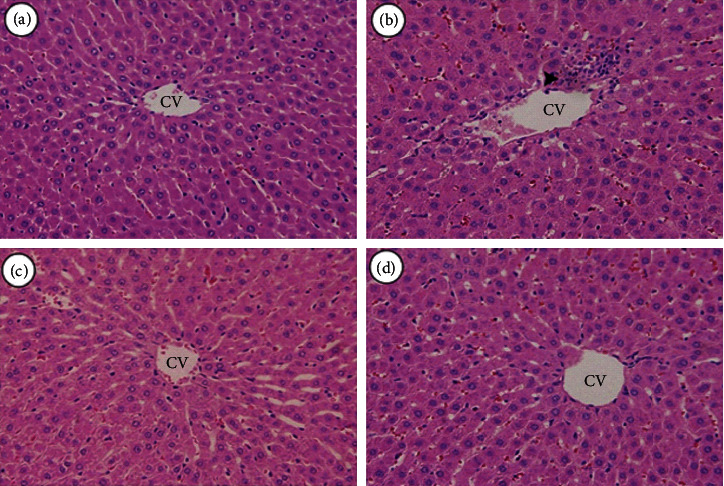
Effect of *L. regularis* (Lr) extract on STZ-induced pathological changes in liver samples as visualized in the histopathological slides stained with H&E (400x). (a) Liver samples from control rats showing normal central vein (CV) and regular hepatic cords. (b) Liver samples from diabetic-untreated animals showing fatty degeneration, dilated central vein (CV), and leukocyte infiltration (arrowhead). (c) Liver samples from Lr (150) group showing partial regenerating hepatocytes in the midzone around central vein with dilatation in hepatic sinusoids. (d) Liver samples from Lr (300) group showing binuclear regenerating hepatocytes with regular central vein.

Semiquantitative histological scoring of liver samples from all groups is presented in Table 1. Histological analysis of the liver sections from control animals revealed normal architecture of hepatocytes. There was a moderate steatosis, fatty degeneration, and inflammatory infiltrating cells such as macrophages and neutrophils in diabetic-untreated animals. Animals treated with *L. regularis* (150 mg/kg) showed partial regeneration of the hepatocytes and mild inflammatory infiltrate. Animals treated with *L. regularis* (300 mg/kg) demonstrated regenerated hepatocytes with minor inflammatory infiltrate (Figure 6).

## 4. Discussion

Diabetic-induced hepatotoxic injury is an important metabolic complication of DM, which might diminish the metabolic function of the liver. It has been suggested that persistent hyperglycemia will trigger oxidative stress and the formation of free radicals, which damage the intracellular integrity and physiological functions. In the present study, it was documented that *L. regularis* protects the systemic and hepatic tissue form the deleterious consequences of hyperglycemia in diabetic rats. Treatment of the diabetic animals with the natural compound markedly improved the altered liver function markers and restored hepatocellular construction and integrity. The reported favorable effects are mediated by the anti-inflammatory and antioxidant properties of the active constituents present in *L. regularis* extract.

In the present study, the STZ model of DM was utilized in experimental animals. After systemic absorption, STZ is up taken by pancreatic beta cells selectively. This action is mediated by glucose transporter type 2. STZ induces alkylation of DNA strands inside beta cells, leading to failure in the physiological function of the cells and sudden insulin deficiency [15]. The resulted hyperglycemia and insulin insufficiency reported in the present study markedly induce damages and injuries in biological systems including hepatic tissues.

Our results showed that the commonly used liver function tests were imbalanced in the serum of diabetic animals. In addition, hyperglycemia impaired the lipid profile markers in diabetic animals treated with STZ alone, which is in accordance with earlier studies [16]. The liver is one of the most common organs vulnerable to harmful effects of hyperglycemia. The resulted hepatocellular damage is mainly due to inflammation and oxidative hepatic tissue injury, leading to elevation of the designate serum levels of ALT and AST. These enzymes characterize hepatocellular damage and used as diagnostic markers in case of diabetic associated-nonalcoholic hepatic steatosis [17]. Furthermore, the consequences of hyperglycemia associated-oxidative stress mainly are harmful to liver. Studies have reported that deficiency in insulin secretion stimulates peripheral free fatty acid lipolysis and their movement to hepatic tissues. At this stage, hepatic uptake of the free fatty acids increases the production of very LDL and triglycerides, leading to augmentation of their serum levels [18].

Insulin deficiency might influence the inflammatory response systemically. In the present study, diabetic-untreated animals exhibited significant high values of inflammation biomarkers in their serum including TNF-*α*, IL-1*β*, IL-6, NO, and PGE-2. Insulin regulates the glucose intake in monocytes and other lymphocytes through insulin receptors [19]. It has been shown that hyperglycemia stimulates the proliferation of bone marrow myeloid progenitors, which leads to enhance monocyte secretion (monocytosis) suggesting monocytes as predominant inflammatory cells in diabetes [20]. Moreover, the reduced hepatic antioxidant capacity and oxidative hepatocellular damage triggered the migration of lymphocytes such as monocytes to the injury sites leading to production of inflammatory cytokines such as TNF-*α*, IL-1*β*, and IL-6, which elevates their hepatic expressions. In addition, caspase-3 levels were upregulated in diabetic animals suggesting the involvement of apoptotic pathways. Collectively, these inflammatory responses have harmful effects on liver tissues as demonstrated histologically. Diabetic-untreated animals showed fatty degeneration and inflammatory cell infiltrates, which was reported previously using the same STZ animal model of DM [12].

Hyperglycemia is a well-known triggering factor for oxidative stress and ROS production in hepatic tissues [7]. Therefore, markers of oxidative stress are regularly elevated in liver tissues of diabetic animals. The generated free radicals attack essential molecules in the cellular membrane lipid bilayer leading to lipid peroxidation, which increase the level of TBARS, markers for lipid peroxidation. Moreover, GSH levels were estimated, in the present study, as it is a well-documented endogenous antioxidant molecule. Glutathione is a tripeptide that has two forms: the reduced GSH form, which scavenges free radicals, and oxidized GSSG form. The ration between the two forms determines the redox status in biological tissues. In hepatic tissue diabetic animals, TBARS values were increased, while GSH levels were reduced indicating oxidative cellular damage. Furthermore, antioxidant enzyme activities, which support the cellular functions and prevent the oxidative destructions, were reduced in hepatic cells. SOD catalyzes the transformation of oxygen radicals (O^2−^) to less harmful species such as H_2_O_2_. CAT hydrolyses H_2_O_2_ in to water and oxygen. GPx, GR, and GST enzymes help in the process of glutathione antioxidant defense against free radicals. They catalyze the transformation of two GSH molecules to GSSG, which is associated with reduction of one H_2_O_2_ molecule to two water molecules. It should be noted that oxidative stress initially increases the antioxidant enzymes activities as a defensive cellular mechanism [21]. Nevertheless, these activities are degraded upon persistent oxidative stress exposure due to the consumption of antioxidant defense and harmful effects of free radicals on antioxidant enzymes themselves [21].

Medicinal plants, containing phenolic compounds such as flavonoids, are known for their antioxidant activities. These effects arouse from the ability of their chemical structures to donate electrons or hydrogen molecules, which accordingly act as free radicals scavenging factor [22]. Treatment of the diabetic animals with *L. regularis* markedly corrected STZ-induced hyperglycemia and insulin deficiency. We reported improved serum levels of glucose and insulin after *L. regularis* treatment, suggesting pancreatic beta cell restorative effects against STZ-induced DNA alkylation and free radical damage. In addition, the reported impaired lipid profile and systemic inflammation were restored in diabetic animals treated with *L. regularis*. These effects might explain the traditional applications of the medicinal plant against DM and kidney diseases [14].

Other species from the family Loranthaceae showed similar antidiabetic, anti-inflammatory, and antioxidant activities. A recent study reported that *Loranthus acaciae* Zucc. might reduce blood glucose levels and downregulate markers of proinflammation and oxidative stress [23]. In addition, *Loranthus micranthus* showed marked aphrodisiac as well as antidiabetic properties, which were attributed to restoring redox status [24]. Interestingly, *Loranthus micranthus* enhanced diabetic-induced damages in liver and kidney tissues and repaired glycolytic flux in STZ diabetic rats [25]. Similar antidiabetic and antihyperglycemia activities of *Loranthus micranthus* extract were demonstrated in alloxan-induced diabetic rodents [26].

In the present study, *L. regularis* markedly corrected the hepatocellular degeneration and inflammatory response to hyperglycemia. Moreover, *L. regularis* reduced hepatic lipid peroxidation and improved antioxidant enzymes capabilities. The antioxidant effects of the traditionally used plant were reported previously. Results of Mothana et al' study revealed that methanol extract of *L. regularis* possesses significant antioxidant and anti-inflammatory effects. Other pharmacological effects demonstrated by *L. regularis* in the same study were antinociceptive and antipyretic effects [10]. In addition, several bioactive molecules were isolated from *L. regularis*, which are flavonoids in nature, including quercetin 3-O-a-L-rhamnopyranoside, quercetin 3-O-b-D-galactopyranoside, and quercetin 3-O-b-L-arabinopyranoside [10]. Another study, conducted by the same group, reported moderate antitumor effects beside the antioxidant and antimicrobial activities of *L. regularis* [11]. Moderate antitrypanosomal effects of *L. regularis* extract were also documented in another study [14].

Two major limitations were countered in this study. Firstly, this study omitted the variability between genders in response to diabetic-induced metabolic changes. The impact of gonadal hormones such as testosterone was not evaluated and correlated with *L. regularis* protective effects. Secondly, this study did not assess the molecular mechanistic pathways that might justify the marked *L. regularis* protective effects. However, findings of the study elaborated the antioxidant and anti-inflammatory roles of *L. regularis* in details, particularly the effect on the antioxidant enzymes and inflammatory cytokines.

## 5. Conclusion

Collectively, this study provided strong evidence that the valuable therapeutic effect of *L. regularis* includes protection against hepatic complications associated with DM. *L. regularis* restored the liver functions and cellular damage in diabetic rats. Furthermore, the ability of the plant to scavenger hyperglycemia-induced free radicals and the downregulation of proinflammatory cytokines production could be suggested as underlining mechanisms. Consequently, further clinical studies could promote the therapeutic application *L. regularis* in diabetic patients.

## Figures and Tables

**Table 1:** Semiquantitative evaluations of *L. regularis* (Lr) extract effects on STZ-induced histopathological changes in liver samples.

Group	Control	STZ	Lr (150)	Lr (300)
Scoring	0.10 ± 0.03	2.10 ± 0.4^*∗∗*^	1.8 ± 0.16	1.4 ± 0.13 ^#^

Data were expressed as mean ± SEM (*n* = 6) and analyzed using one-way ANOVA followed by Student–Newman–Keuls as a post hoc test.^*∗*^Control *vs* STZ group; ^#^STZ *vs* Lr (150) or Lr (300). *P* values are considered significant when ^*∗*^ or  ^#^*P* < 0.05, ^*∗∗*^ or  ^##^*P* < 0.01, and ^*∗∗∗*^ or  ^###^*P* < 0.001.

## Data Availability

The datasets generated and/or analyzed in the present study are included in the manuscript.
